# Supplementary figures and images for: Budgerigars have complex sleep structure similar to that of mammals
Source: PLoS Biol. 2020 Nov 17;18(11):e3000929. doi: 10.1371/journal.pbio.3000929 (PMC7707536; doi:10.1371/journal.pbio.3000929)

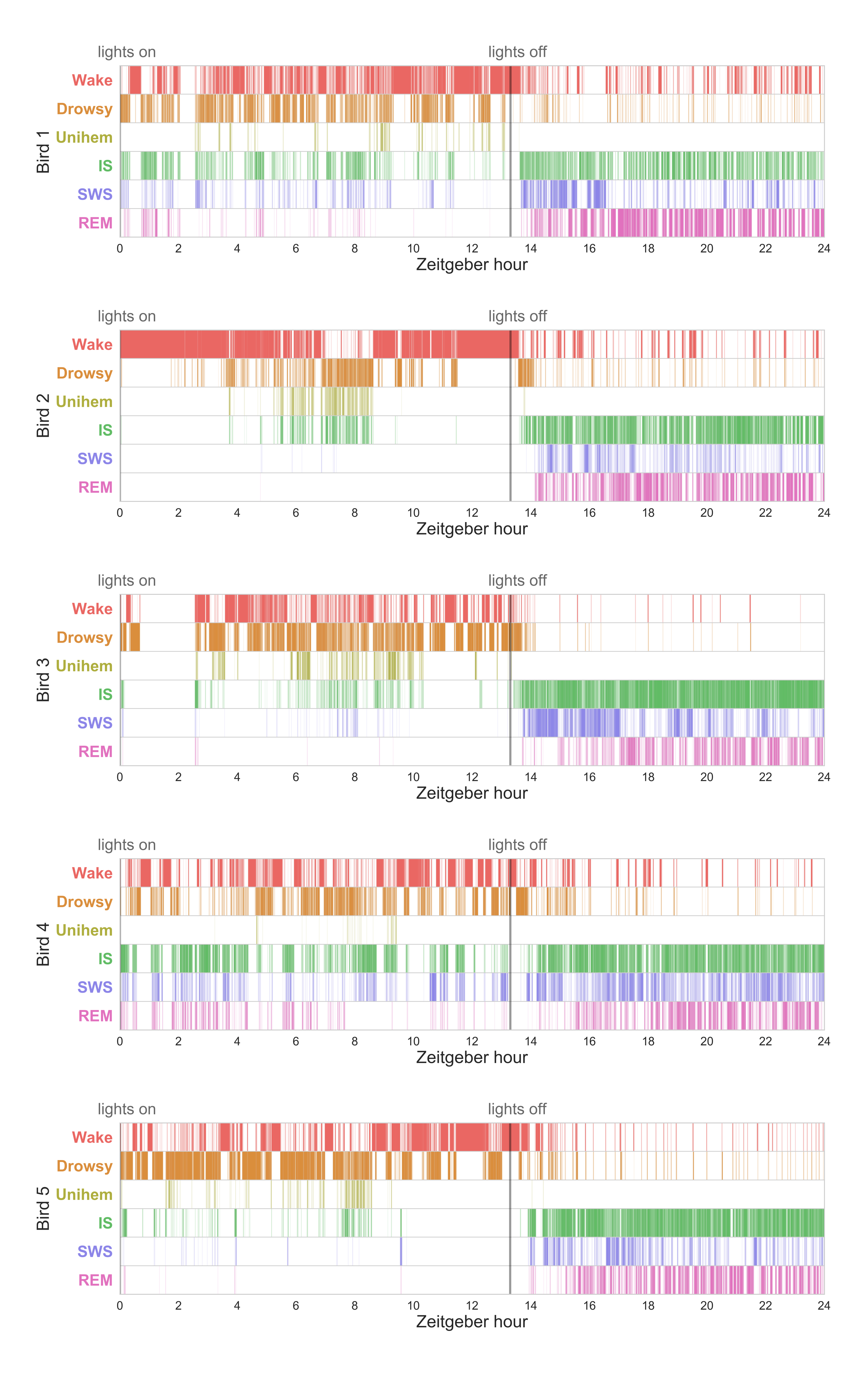

Supplement: S1 Fig — Rows correspond to stage; each tick mark represents one 3-s epoch. Vertical gray lines indicate lights on and off. White gaps starting at approximately 2 h for Bird 1 recording and approximately 30 min for Bird 3 recording indicate missing data due to bird moving out of frame (Bird 1) or technical problems with video recording (Bird 3). Each individual showed somewhat unique sleep patterns, with Birds 1 and 4 exhibiting more and slightly deeper daytime sleep than other birds. In general, sleep was much more consolidated at night; daytime sleep was fragmented and most prevalent in the afternoon. The patterns of REM increase and SWS decrease across the night are also visible here. Raw scores are provided in S2 Data. IS, intermediate sleep; REM, rapid eye movement sleep; SWS, slow wave sleep; Unihem, unihemispheric sleep. (PNG) [file pbio.3000929.s002.png]

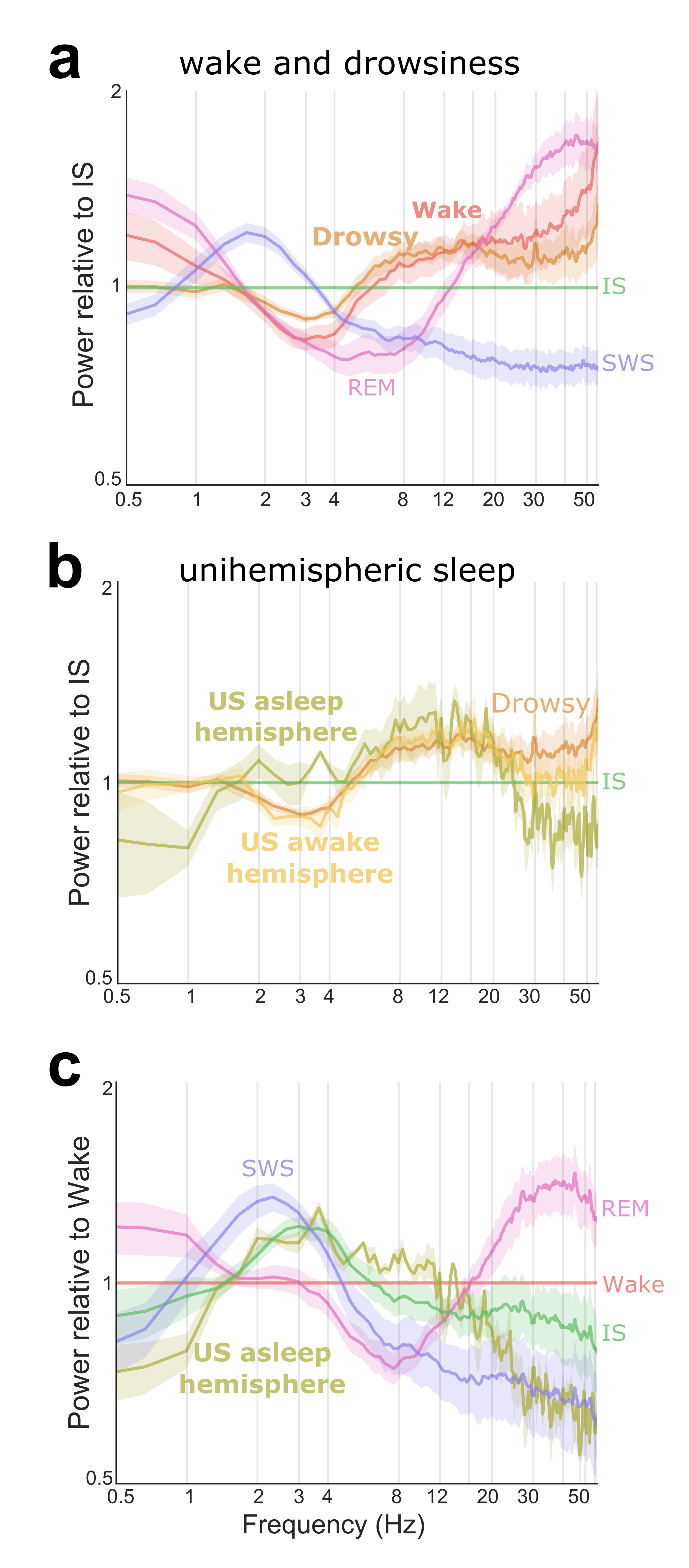

Supplement: S2 Fig — (a) Spectra of Wake and Drowsy compared to REM and SWS, plotted relative to IS. The pattern of REM is markedly similar to Wake and Drowsy. There were no statistically significant differences between Wake and Drowsy. The qualitatively slightly lower gamma and slightly higher delta during drowsiness are consistent with the description of drowsiness as an awake state with some (NREM) sleep-like characteristics. (b) Unihemispheric sleep, plotted relative to IS, with Drowsy shown for comparison. EEG from the awake hemisphere (corresponding to the open eye) is contrasted with the sleeping hemisphere (closed eye). Due to the small amount of US detected in this study, sufficient artifact-free US data was collected from 5 channels and 4 channels, respectively, from 3 birds. The awake hemisphere is most similar to Drowsy. (c) Unihemispheric sleep (sleeping hemisphere), plotted relative to Wake, with REM, IS, and SWS shown for comparison. The sleeping hemisphere is most similar to IS, except for very low power in the gamma band most similar to SWS. Data are provided in S1 Data (under Fig 4A–4C). EEG, electroencephalogram; IS, intermediate sleep; NREM, non-REM; REM, rapid eye movement sleep; SWS, slow wave sleep; US, unihemispheric sleep. (PNG) [file pbio.3000929.s003.png]

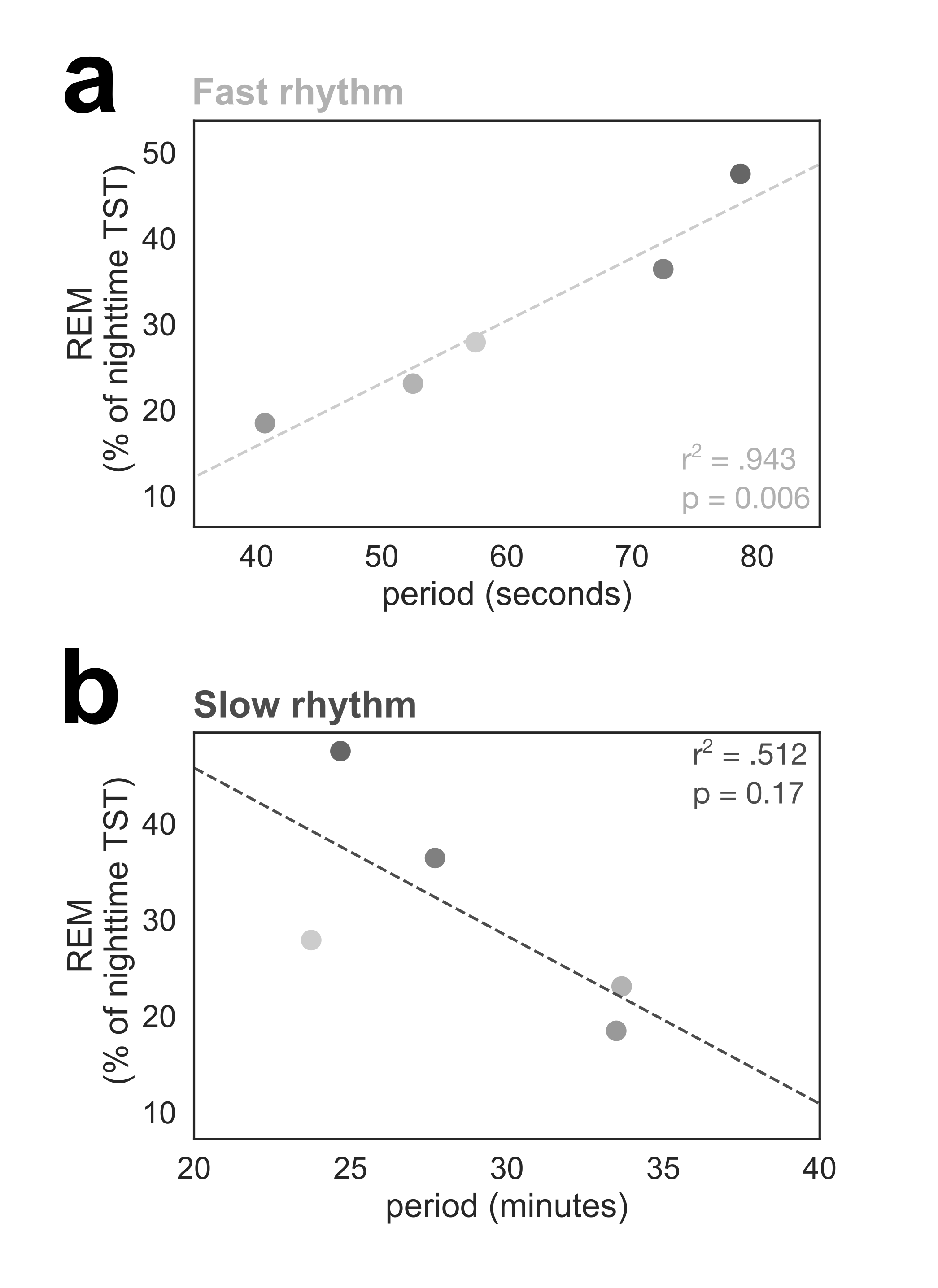

Supplement: S3 Fig — The color of each dot corresponds to bird identity, with Bird 1 in the darkest shade and Bird 5 in the lightest. Dashed line, line of best fit as determined by least-squares linear regression. (a) Fast rhythm (average period = 60 s); a positive correlation with REM was observed. (b) Slow rhythm (average period = 29 min). Data are provided in S1 Data. REM, rapid eye movement sleep; TST, total sleep time. (PNG) [file pbio.3000929.s004.png]

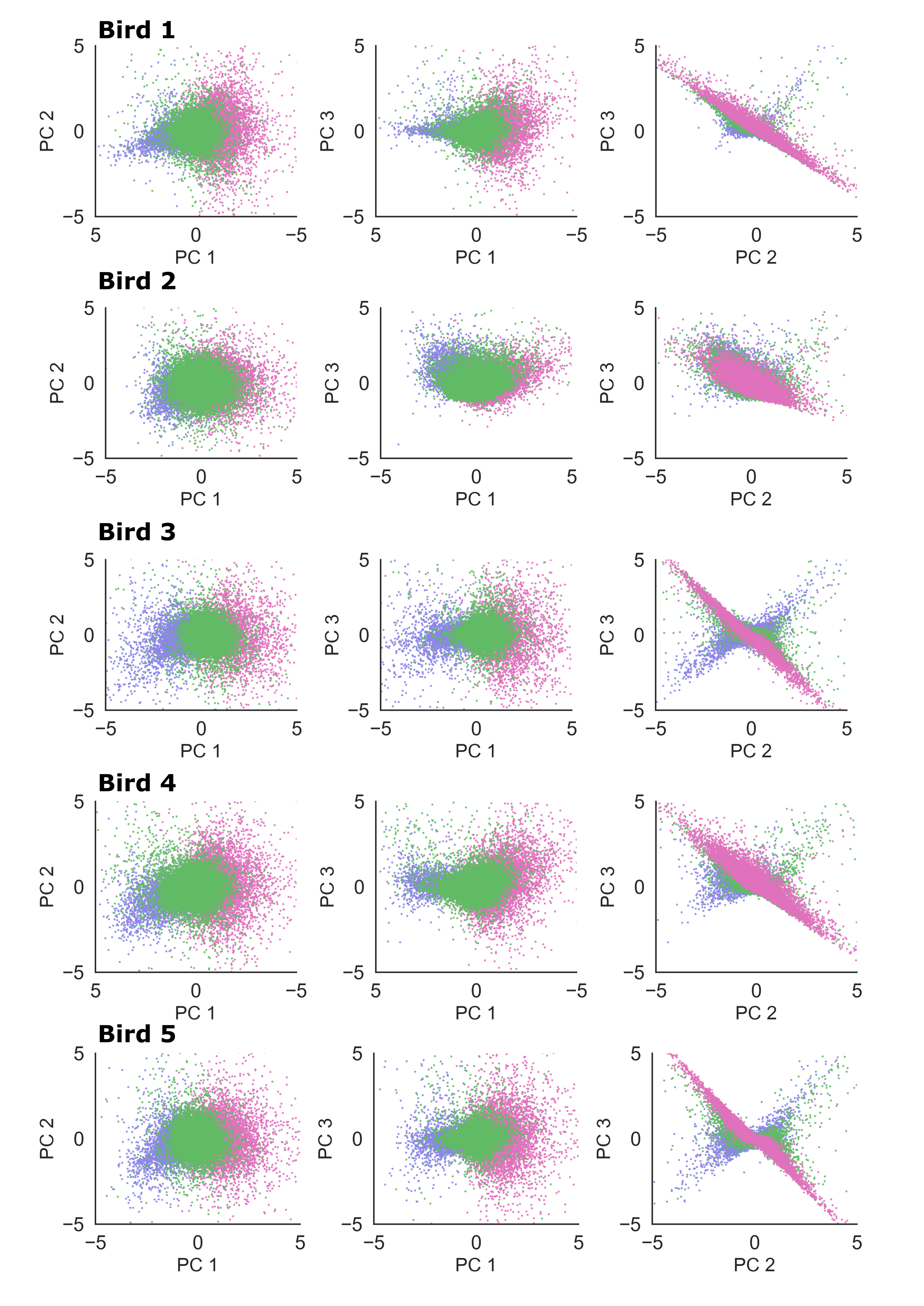

Supplement: S4 Fig — PCA was performed on the five-dimensional space of log(delta), gamma/delta, gradient of delta, gradient of gamma/delta, and standard deviation, as described in Fig 8. Each column shows a different combination of the first 3 principal components. Note that for Birds 1 and 4, the first PC is plotted from positive to negative to facilitate comparisons between all 5 birds. The first 2 columns show IS clustering in the center of PC1, with SWS and REM fanning out to either end. The rightmost column shows SWS and REM falling along 2 orthogonal planes, with IS forming the transitional area in between. This pattern was least robust in Bird 2, which had the lowest percentage SWS. Purple denotes REM, blue denotes SWS, and green denotes IS. Data are provided in S1 Data. IS, intermediate sleep; PC, principal component; PCA, principal component analysis; REM, rapid eye movement sleep; SWS, slow wave sleep. (PNG) [file pbio.3000929.s005.png]

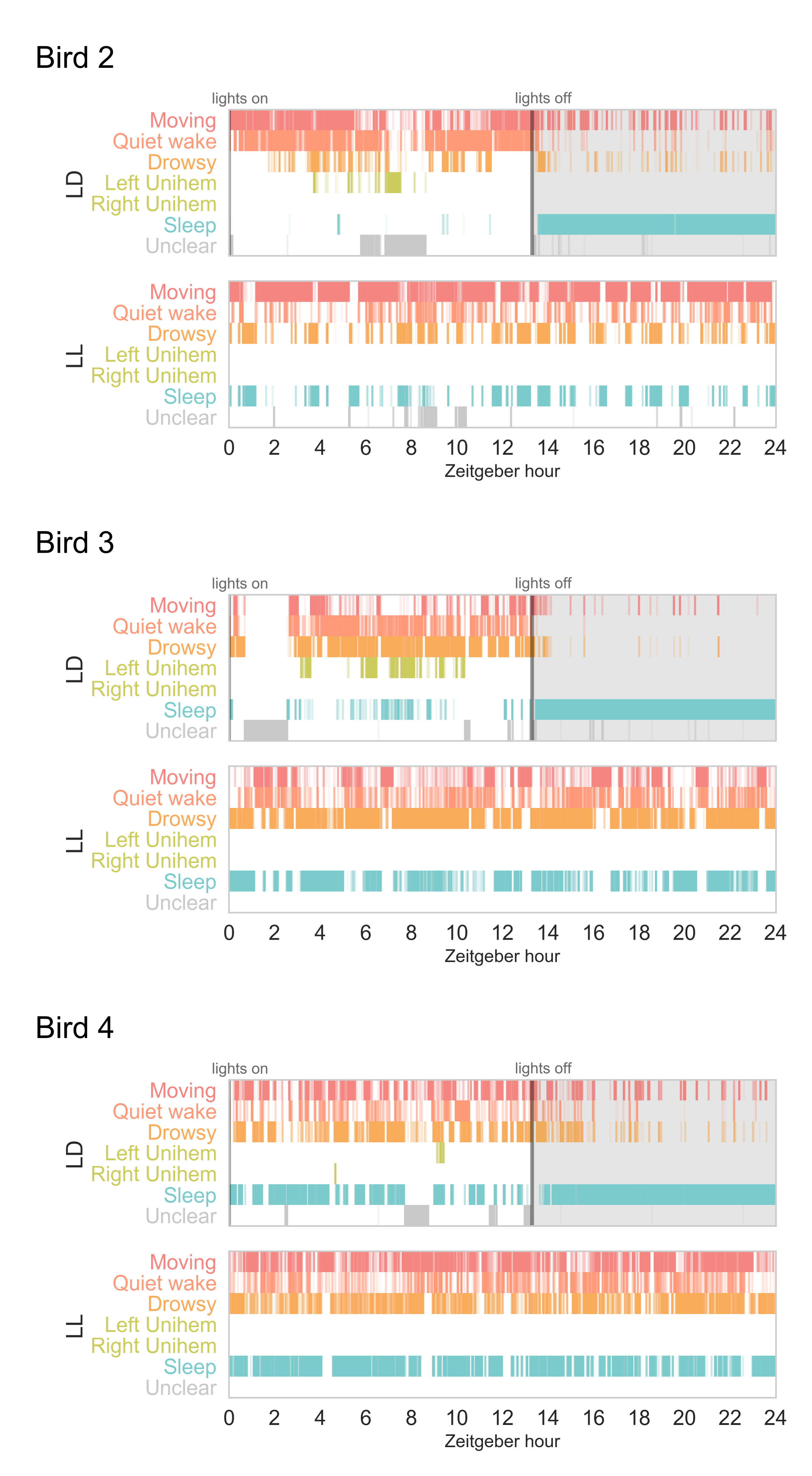

Supplement: S5 Fig — LD, baseline 13:11 light:dark cycle; LL, constant bright light. Rows correspond to state as determined by video scoring; each tick mark represents one 3-s epoch. Vertical gray lines indicate time of lights on and off in baseline LD; gray shading indicates dark period. Differences in unihemispheric sleep in Birds 2 and 3 were likely due to altered camera positioning in LL (see Methods). In LL, sleep was reduced, fragmented, and spread out across the day. Raw scores are provided in S2 Data. LD, light/dark; Left U, left-brain unihemispheric sleep; LL, constant light; Right U, right-brain unihemispheric sleep. (PNG) [file pbio.3000929.s006.png]
