# Supplementary material for: Budgerigars have complex sleep structure similar to that of mammals
Source: PLoS Biol. 2020 Nov 17;18(11):e3000929. doi: 10.1371/journal.pbio.3000929 (PMC7707536; doi:10.1371/journal.pbio.3000929)
Supplement: Attachment — Submitted filename: Canavan budgie ms - response to reviewers.pdf [file pbio.3000929.s009.pdf]

We are grateful for the careful reading, the many comments and the suggested analyses the reviewers provided for us. We have made extensive responses to these, which has very substantially improved the reporting and expanded the description of the results. We believe the end product is a greatly improved manuscript and are excited by these changes, prompted by the review process. The revised paper has three additional figures, one additional supporting figure, and now has one supporting video.

Reviewer 1

1.0

The authors provide a thorough analyses on sleep in 5 Budgerigars under light-dark conditions and under constant light conditions. The results show that sleep in this bird species, like that of song birds and other parrots, seems not that different from sleep in mammals. The authors put their results in an evolutionary context.

Although impressed with the detailed analyses presented and several new aspects of sleep that were discovered, the study remains a purely descriptive study in a very small number of individuals, partially confirming a 30-year older sleep study performed in this same species. The study did not yield new insights into the question concerning evolution of mammalian-like sleep features, as the separation and alternation of clear REM sleep and NREM sleep states (the main sleep phenotype that has prompted recording sleep in monotremes, basal bird, reptiles, and even fish), was already observed in the older parrot studies.

We respectfully disagree with this characterization. The previous budgerigar study [1] found 5% REM, 25% TST, did not describe an intermediate sleep stage, did not describe any patterns in slow wave activity, and characterized the birds as polyphasic sleepers. In contrast we observed 5x the amount of REM and 2x the amount of TST; characterized a prevalent intermediate sleep stage as well as unihemispheric sleep; found homeostatic-like and ultradian patterns in SWA, systematic ultradian variation in the amount of REM, a consistent transition pattern between the sleep stages, two ultradian rhythms, and a firmly diurnal (rather than polyphasic) sleep pattern. These are significant advances over the older study. These data support the conclusion that budgerigar sleep shares many similarities with mammalian (including human) sleep, the opposite of the conclusion of the previous study. This motivates a re-evaluation of budgerigar sleep from an evolutionary perspective. We also demonstrate that several of the erroneous conclusions of the older study arose from technical limitations of that era (requiring conducting the analysis under constant light conditions) which we could address using infrared video observation.

In totality, these are significant departures from the conclusion of Ayala-Guerrero et al., and we note our findings fit into and expand a broader pattern of modern reinterpretation of sleep in birds (and reptiles) that is emerging. We would also suggest there remains uncertainty as to the sleep structure in basal birds and hence the evolution of complex sleep in birds, given that somewhat conflicting results have been reported in tinamous [2] and ratites [3], and with both these groups parts of a radiation

distinct from basal birds. We discuss this in the paper in relation to our results, helping to provide more context for the evolution of sleep in birds.

We are also unclear in what sense our study is purely descriptive, given that we described sleep in unmanipulated birds, formed a hypothesis as to why our results differed from the prior study of budgerigars, and then conducted an experimental manipulation to test that hypothesis. Resolving that discrepancy gives additional confidence to our results.

Finally, our sample size is not small as compared with other studies. Reviewing 47 papers on bird sleep from 1964 to 2020, the mode number of subjects was n=5 (Table 1). Furthermore, our approach was labor intensive, requiring manual scoring of both the video recordings and then the polysomnography data, prior to the signal processing and statistical analysis. This limits the number of individuals evaluated but provided for a deep analysis of the sleep structure of each one. Taking this approach, we achieved reliable results supported by statistical analysis and a number of worthwhile novel observations. By what objective criterion is the sample size "very small" relative to the results that we report? Certainly, we acknowledge that a larger sample size would likely have provided more power in the statistical tests to evaluate changes in sleep structure comparing LD and LL birds (for example). That is the trade-off in our design, but it was made knowingly to enhance the positive attributes described above. Most of the results we observed would not have been possible with a less thorough analysis.

Table 1.

Reference	Year	Species	Age	N
[4]	2020	Starling	Adult	12
[5]	2018	Pigeon	Adult	6
[6]	2017	Barn owl	Nestling (36-49 days)	42
[2]	2017	Tinamou	Adult	3
[7]	2016	Frigatebird	Adult	9
[8]	2014	Quail	Adult	8
[9]	2013	Barn owl	Hatchling	66
[3]	2011	Ostrich	Adult	6
[10]	2008	Zebra finch	Adult	5
[11]	2008	Pigeon	Adult	5
[12]	2008	White-crowned sparrow	Adult	6
[13]	2006	Pigeon	Adult	5
[14]	2004	White-crowned sparrow	Adult	8
[15]	2003	Turkey	Adult	10

[16]	1996	Blackbird	Adult	5
[17]	1994	Pigeon	Adult	7
[18]	1994	Mallard	Adult	5
[18]	1994	Zebra finch	Adult	6
[19]	1993	Blackbird	3 adult, 2 juveniles	5
[20]	1989	Rook	Adult	9
[20]	1989	Magpie	Adult	6
[1]	1989	Budgerigar	Adult	4
[21]	1988	Orange-fronted parakeet	Adult	5
[22]	1988	dove *Zenaida asiatica*	Adult	5
[23]	1988	Pigeon	Adult	6
[24]	1987	Rook	Adult	6
[25]	1987	Chicken	Adult	5
[26]	1986	Emperor penguin	Adult	4
[27]	1986	Jackdaw	Young adult	8
[28]	1986	Starling	Adult	4
[29]	1985	Starling	Adult (2 yrs old)	4
[30]	1985	Goose	Adult	7
[31]	1984	Little penguin	Adult	5
[32]	1983	Ringneck dove	Adult	4
[33]	1976	Chicken	Day of hatching	2 to 7 per group
[34]	1975	Chicken	3-4 hrs, 8 hrs, and 3-4 days posthatch	4 in younger two groups; 3 in older group
[35]	1974	Chicken	1 day to 4 months	1 day (n=3)<br>1 week (n=7)<br>1 month (n=4)<br>4 months (n=3)
[36]	1973	Tawny owl	Adult	2
[37]	1973	Chicken	Adult	17
[38]	1973	Chicken	9 - 15 weeks	35
[39]	1972	Burrowing owl	Adult	4
[40]	1972	Pigeon	Adult	3
[41]	1972	Pigeon	Adult	8
[42]	1970	Hawk	Adult	1
[42]	1970	Falcon	Adult	1
[43]	1969	Chicken	8-15 days	11

[44]	1966	Pigeon	Adult	15
[45]	1965	Chicken	6-9 months (males) and 8-10 months (females)	15
[46]	1964	Chicken	Adult	7
[47]	1964	Pigeon	Adult (> 1 year)	3
[47]	1964	Chicken	Juveniles (2-3 months)	4
[47]	1964	Chicken	Chicks (0-3 weeks)	24
[47]	1964	Chicken	Adult	1

1.1

The choice of recording this particular species is unclear. A bigger parrot species could have been chosen allowing the use of wireless system as is now standard in bird sleep studies, which in an animal that flies seems a great plus over a tethered system.

We chose to study budgerigars because they are a small and widely available species of parrot that is relatively easy to care for in a lab setting, and as such are vastly by far and away the most widely studied parrot species in psychology and neuroscience [including but hardly limited to 48-67]. Thus, by choosing budgerigars our current results are well positioned to maximize their impact on knowledge in the field. Indeed, given that there are only two studies characterizing sleep in a parrot species [1,21] it would have diluted the interpretation of our results had we chosen a third species to study, and it would have diluted the insight we achieved as to reinterpreting the prior results. Finally, given that many species of larger parrots are endangered and are highly intelligent, we firmly believe it was both prudent and ethically responsible to have started this research thrust with budgerigars. We or other laboratories are now better informed as they decide if and how to extend this work to more precious animals.

We too applaud the use of miniature data loggers as reported for ostriches [3], barn owls [9], frigatebirds [7], tinamous [2], pigeons [5] and starlings [4]. Most (or perhaps all) of these studies arise from the Rattenborg group, and so it is an unsupported claim and, in our opinion, inaccurate to state that this has become the standard in the field rather than in one laboratory. Furthermore, like any technique the use of miniature data loggers has strengths and limitations. These devices remain too large to be used in budgerigars (and too large for the great number of small bird species). More importantly, there is no objective evidence we are aware of that a tether approach as we used here precludes achieving biologically meaningful results. This approach has been a mainstay for perhaps 50-100 publications or more in birdsong production work. This approach was successfully used by us in a prior study of zebra finch sleep [10]. In our hands, with approximately 25 years of experience with this approach [see 68], once birds carrying tethers adapt to the new environment, they exhibit normal sleep behavior including occasional intervals of sleeping with beaks between the wings, which is an

excellent indicator of comfort. We have observed this in a number of species. Tethered budgies occasionally showed such sleep postures, too.

1.2

Other comments concern the surprising lack of recording muscle tone which for mammalian sleep studies would be inadmissible although the EOG and video recording leave no doubt on the sleep states identified.

We agree that it would be unusual for a mammalian sleep study to omit EMG. However, this is a relatively common practice in avian sleep studies due to the lack of consistent muscle atonia/hypertonia during REM [4,7-10,12,14,15,18-20,31,35,36,40]. There are several bird species that are possibly exceptions to this rule, e.g. ostriches [3], or partial exceptions e.g. geese [30], but in avian studies where EMG has proven useful, it seems to be primarily used to distinguish wake and REM, e.g. in situations where video recording is not available [3,27,30,35]. Parrots, however, were not previously reported to have REM hypertonia [1,21]. Our observations were consistent with this; while we saw occasional head drooping, twitching, etc. during sleep in budgerigars, this was not a sensitive marker of REM and could occur in other sleep states. Thus, we made a well-reasoned decision supported by extensive prior studies to forgo EMG recordings.

We appreciate the comment of the reviewer and have added a section clarifying our rationale for not collecting EMG (in Methods, 'Surgery') and have also expanded on our observations of head drooping and twitching during sleep (in Results, 'Characteristics of budgerigar sleep').

1.3

The large amounts of REM sleep observed were put into a context of learning but REM sleep is also associated with stress that might be high in this highly social animal kept in isolation.

Most sleep studies, like ours, are conducted in isolation to avoid the addition of multiple confounding variables e.g. differences in specific relationships between individuals, interactions between individual sleep patterns (e.g., if one bird wakes up at night, this will likely wake up the other bird). Indeed, isolation from conspecifics is nearly ubiquitous in avian sleep studies. Importantly, this includes the two previous parrot studies [1,21]. The birds in Ayala-Guerrero et al were isolated for 1 or 2 weeks before recording, while ours were isolated for 9 - 15 days. Thus, an increase in stress from isolation does not explain the differences between our results and those of Ayala-Guerrero et al.

We attempted to mitigate the effects of social isolation by housing birds together until shortly before surgery; providing mirrors, which provide some social stimulation to budgerigars [69]; and opening sound box doors when not recording, allowing birds to

interact vocally. We thank the reviewer for this comment as well and have added these details to the Methods (section ‘Animals’) of the paper.

1.4

Also the presence of 2x more REM sleep in one bird compared to the other 4 does not convince that the REM sleep measured is a robust finding.

Respectfully, this is an inaccurate description of our results; perhaps the reviewer is confusing the amount of REM sleep with the duration of REM sleep episodes, which were about 2x higher for one bird. The amounts of REM in the five individual birds, in % of TST across 24 hours from lowest to highest, were 16.2%, 20.4%, 25.4%, 32.0%, and 38.6%. The values are similarly distributed for REM at night only, or REM calculated as a % of total recording time. The distribution of REM at night, during the day, and across 24 hours can be seen in Fig 2c and Fig2e-g; all these values will be available to readers in the supplementary data, and prompted by the reviewer’s comment we now include a subset of this individual bird data in Table 1. Thus, there was no bird with 2x the amount of REM of the others. The other analyses reported in the paper help to convince that this is a robust finding, including the spectral analysis, slow wave analysis, automated detection of eye movements, and clustering analysis/automated scoring. We now also report a correlation with nighttime REM% for the fast rhythm (Figure S3), giving further confidence in the REM scoring.

Furthermore, the amount of inter-individual variation is consistent with other sleep studies. Compare with Low et al [10], in which the % REM of the 5 zebra finches ranged from 15.5% - 34.3%. In that paper, characteristics of REM such as duration of episodes showed similar variation:

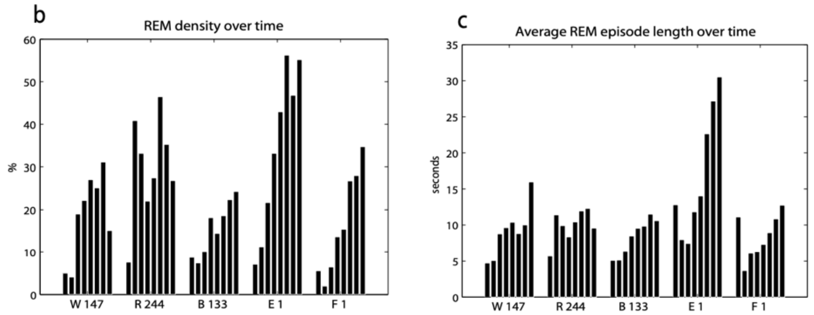

In human sleep, considerable inter-individual variation is also apparent [e.g. 70]. This including a range of % REM spanning an approximately 2x difference at any given age, for example in this figure from a 2004 meta-analysis [71].

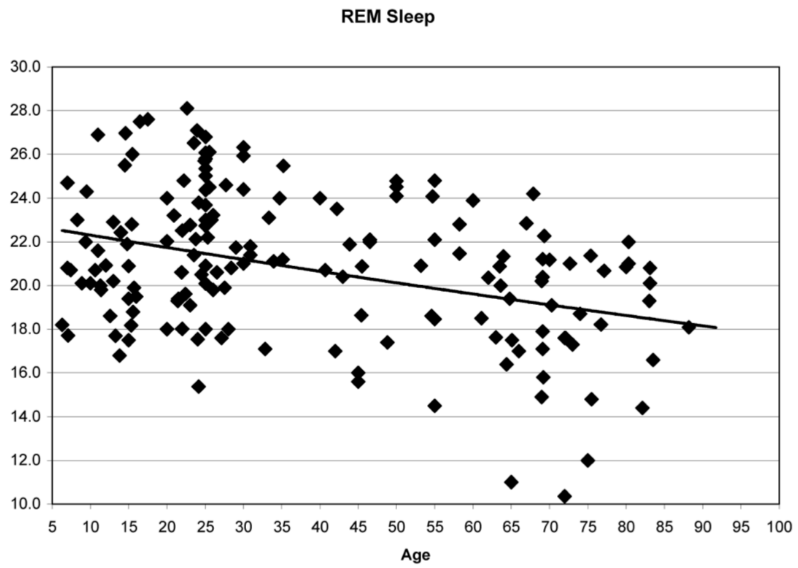

Thus, the variation in budgerigars we observed is consistent with a broad range of sleep research spanning many different animal species, and humans.

1.5

The authors equate SWS with SWA (slow wave activity) in the discussion. Although SWS and SWA are related measures, they are not the same.

Minor point, we did not explicitly equate SWS with SWA, we discussed previous results on SWA because they are consistent with our current results re. SWS. Nevertheless, we agree with the thrust of the reviewer comment and have clarified the language surrounding SWS and SWA in the discussion.

1.6

If the authors aimed at addressing homeostatic regulation of NREM sleep slow waves, why didn't they quantify SWA in NREMS?

As part of the analyses in Figure 5a, we measured the average power in the delta band for each epoch across the 24-hour day, which is equivalent to the SWA. We have clarified this in the text of the Results. We have also added a figure showing patterns in average hour-by-hour SWA across the 24-hour day (Figure 4d).

1.7

Related to this, since slow-wave content in NREM sleep is a continuum, what made the authors decide that SWS and IS are two discrete states?

We agree that NREM contains a spectrum of SWA, with IS and SWS reflecting a division of this spectrum into two parts. We have clarified the language in the discussion

to reflect this. Note that IS tends to be resistant to spectral manipulations that collapse the distributions of SWS or REM (Figure 8).

The SWA in human NREM is likewise a continuum, but the distinction between N2 and N3 is still used consistently, has lasted through several revision of the sleep staging system [72,73], and has helped the field enormously. Given the presence of similar NREM structure now shown in several species of birds, we believe that the IS/SWS distinction would similarly benefit the avian sleep literature. This classification helps to highlight structure not traditionally focused on in the avian sleep literature (for example, Figures 3a-d directly compare how sleep structure appears when NREM is separated into IS and SWS and when it is not). It might contribute to identifying differences between individuals. If more widely adapted, this classification could be used to understand species differences, e.g. in meta-analyses [74]. More generally, separating IS from SWS brings the animal research further in line with human research, allowing comparisons and insights to more easily flow between the respective fields.

Reviewer 2

This is a very interesting and thorough study that adds new data to the story of sleep phylogeny. In particular the finding of so much REM sleep in this bird species is quite fascinating. I only had a few comments.

2.1

1. The authors discuss sleep homeostasis in this species, but its not clear if changes in delta wave (or slow wave) activity (SWA)were measured across the 24 hour day. In mammals, EEG SWA is more finely regulated by prior wake time than other sleep metrics (e.g. sleep time). Therefore it would be useful to include this analyses.

We agree that measuring SWA over the 24-hour day would be useful, and so we have added a figure showing the average SWA per hour (Figure 4d). As per the reviewer's suggestion, we have compared this to total sleep time per hour. Although not a direct test (it was not a goal of our study), this result is consistent with the idea that prior wake time is an important determinant of SWA; we have added a discussion of this in the text.

Figure 5a shows the total delta power in each epoch, for one representative bird, over a night of sleep. We have clarified that this is equivalent to SWA in the text and the labeling of the figure.

2.2

2. I have two points about the unihemispheric sleep. In figure 1, unihemispheric sleep looks more like IS than SWS, but its not clear. Spectral analyses of this state could help in this regard, as is done in Figure 4.

We have added spectral analyses of the two hemispheres during unihemispheric sleep (US; Figure S2b-c)**.** This indeed supports the reviewer's observation of Figure 1, in which the sleeping hemisphere during US resembles IS. We have also expanded our description of US scoring to clarify how it relates to our scoring of IS and SWS.

2.3

The other question is, was there ever unihemispheric REM sleep?

The prospect of unihemispheric REM is very interesting, but we did not see evidence for it. Such a state, however, would be very difficult to distinguish from drowsiness with a momentarily closed eye. A more specialized study, specifically designed to capture US, would likely be needed to rule the possibility of unihemispheric REM in or out. As discussed in the methods, we were limited in our ability to characterize US in these birds given their mobility at night which often precluded observing both eyes simultaneously. In conjunction with the similarity of US to drowsiness, this limited the amount of US we could confidently identify.

Reviewer 3

The study by Canavan & Margoliash thoroughly characterizes the sleep architecture in budgerigars. They find that the sleep pattern in these birds is highly complex: The recorded animals show a lot of REM sleep (more than reported in any mammal), sleep periods are extremely short, rapidly alternating and the brain state is thus constantly changing on a time scale of 10s of seconds. Nevertheless these birds show a distinct ultradian rhythm (~30 min oscillation). As the present study adds important data to our understanding of sleep in birds and the evolutionary origins, it is highly significant and possibly explains contradicting results from previous work on sleep in parrots. However, more careful analysis is required to demonstrate that the defined sleep stages indeed represent distinct brain states and that the applied criteria for sleep scoring thus correctly describe sleep in budgerigars.

Major Concerns

3.1

1. Comparison of the power spectra of the three scored sleep stages (SWS, IS, REM) in Fig. 4c shows that the three states are quite similar in term of their frequency composition and questions to what extent different manual scorers or automated algorithms (especially when relying on thresholds) will arrive at the same results. Overall, the robustness of the presented data strongly depends on to what extent the defined sleep stages correspond to distinct electrophysiological states. In case the different sleep stages are not distinct, but rather lie along a continuum of slightly varying states, the scoring will likely depend on the actual criteria of the manual/automatic classifier. To convincingly demonstrate that the defined stages correspond to separate states that can be clearly determined either manually or automatically, the authors should perform some form of clustering and/or dimensionality reduction analysis (e.g. PCA as previously performed for zebra finches Low et al., 2008).

We have addressed this point in several ways. First, we have altered Figure 4 to more clearly display the power spectra and add context. We now included the averaged power spectra of wake for context. We have also plotted each stage relative to either wake or IS, allowing us to show the SEMs and all frequencies of interest on a single plot. Furthermore, for the overall power spectra (Figure 4a-c) we have also more aggressively removed epochs containing eye movement artifact, having found that such artifacts contaminate the 0 – 1 Hz range. Upon reviewing Kis et al, 2017 [75], we have also slightly adjusted our normalization technique (Figure 4a-c) to use total power from 1 – 55 Hz rather than 0 – 55 Hz. Please note, these updated analyses changed by small amounts many of the numerical values reported for the statistical tests but did not alter the significance or lack thereof of any of the results.

Secondly, we have performed clustering analysis to automatically score sleep states, based on a slightly modified version of the algorithm used in Low et al 2008. The REM/NREM concordance found between manual and automated scores was very similar to that reported in Low et al (the SWS/NSWS concordance was not possible in the zebra finch study, as manual scores were limited to REM and NREM only). We additionally plotted sleep epochs in spectral feature space and in principal component space, replicating the patterns seen in zebra finch sleep (Figure 8).

Finally, we have clarified the language in the Discussion surrounding IS vs SWS: we view these states as two ends of a continuum of SWA within NREM, similar to the way in which human N2 and N3 are classified.

We thank the reviewer for the comment, which provided insight for us to improve the paper.

3.2

2. The logic of sleep state transitions remains unclear. In other words, are their regularities in brain state transitions or do brain states follow each other quite randomly?
The authors could calculate for example a Markov matrix or graph with nodes (states) and edges (transition probabilities) showing how likely transitions between pairs of brain states are. For example can REM sleep only be entered from SWS or also from IS. What stage typically follows REM sleep etc.?

We have added this suggested analysis, displayed in the form of both chord diagrams and the transition matrix (Figure 3 e-h). The pattern of transitions across birds was quite consistent; for example, transitions both into and out of REM were primarily through IS. We also show the REM latencies (time from sleep onset to REM onset) in Figure 9c, underscoring the point that REM tended to occur later in sleep episodes. These provide more insight and additional confidence in the results, here too prompted in response to the reviewer comment.

3.3

3. The conclusion that sleep recordings under LL condition are similar to those in a previous study [23] is only based on a single animal ("14.6% TST in one bird compared to 25.15%, an average across 4 animals, reported in [23]"). To draw this conclusion the authors should also include the remaining animals (recorded under LL) into their analysis. According to Methods (p.36, ll. 725-728) PSG data were recorded for all three animals under LL. What are the TST and REM sleep percentages for all birds and how to they compare those reported in [23]?

We have now scored video data from all 3 birds in LL, showing a significant reduction in TST by nearly half (Figure 9b) and a considerable decrease in the duration of sleep episodes, suggesting fragmentation (Figure 9c).

We have added spectral analyses of the LL sleep PSG data to objectively quantify changes from LD to LL. This includes analyses of the SWA (Figure 9f-g) and nPeaks, which measures high frequency activity that is typically associated with REM (Figure 9h-i).

3.4

4. Does the analysis for the recordings under LL (data shown in Fig. 3 and values reported on p.13, ll. 255-260) also include PSG recordings or only video monitoring? The results in Fig. 3 and p. 13 are intended to resolve a conflict with a previous study [23]. In case, only video analysis was performed the authors have to validate that video analysis provides similar results as sleep scoring based on EEG/EMG/EOG.

We agree this is an important point, thank you for the comment. Video was the primary means of distinguishing wake from sleep, as birds do not show a clear alpha rhythm. Thus, during PSG scoring only a small number of drowsy epochs (2.88% ± 0.57%) were changed to sleep and conversely, only a small number of sleep epochs were re-classified as drowsy (2.64% ± 0.51%). The overall TST scored by video was very similar to TST scored by PSG (video, 47.09% ± 2.71%; PSG, 47.35% ± 2.67%). Video TST was higher than PSG TST in 2 birds and lower in 3 birds, for an absolute average change of 1.45% ± 0.38% in TST per bird. We have added these values to the Results under "Effects of constant light".

3.5

5. Do the ultradian rhythms (Fig. 6), at least to some extent organize, the expression of NREM and REM. E.g. does REM (or other states) occur at a particular phase of the (1 min) rhythm.

We have incorporated this analysis into the paper (Figure 6e-g; examples in 6b). We now show that the fast rhythm (~60 s) separates REM and NREM, while the slow rhythm (~30 min) separates all three stages with REM at the peak and SWS at the trough.

Minor Concerns

3.6

Fig. 1

Why does the number of EEG channels in h,i differ from that in the other subfigures?

For Figure 1 b-g, we showed data from one bird to allow for the clearest comparison between different wake/sleep states. The data in Figure 1h and 1i are additional examples from a different bird. In addition to the specific phenomena displayed in 1h-i, we wished to provide a second example of REM, IS and SWS, allowing comparison of sleep states across two individuals. The bird in Figures h-i happened to have more artifact-free EEG channels. We now state this in the figure legend. Showing all the channels we analyzed for a given bird better represents graphically the data we worked with, while avoiding generating unwarranted concerns about cherry picking which traces we choose to display.

3.7

Fig. 2e,f,g
Please connect the dots with lines to show which data points belong to the same animal

We have now added lines in greyscale to (Figure 2c and e-g), with different shades corresponding to individual birds. These colors have now been carried throughout subsequent figures (e.g. Figures 3, 6, 9). This should allow the reader to follow a single individual across multiple plots.

3.8

Fig. 4
Add bar plots representing frequency of sleep state episodes to more thoroughly represent sleep architecture.

These have been added as Figures 3c-d.

3.9

p. 4, l. 64
")" missing

Done.

3.10

p. 19, ll. 400-401
"Eye movement content during REM was distributed broadly with a pronounced peak at 0."
What means "peak at 0" in this context?

By "peak at 0" we meant that the largest number of REM epochs had no eye movement. We have reworded this awkward language.

3.11

p. 12, lines 237 - 240
"For the one bird (a male) where we conducted manual video scoring (Figure 3a), this revealed 14.6% TST in LL; this bird had 38.8% TST at baseline. This is consistent with the difference in TST between previous work in LL (25.15% of time) [23] and our study in LD (47.35% ± 6.68% of recording time)."
What means "at baseline" in "38.8% TST at baseline"? How does the value compare to the value 47.35% reported in line 240? Is this an average across all birds?

We have clarified that "baseline" refers to the data collected in normal LD conditions. This section has changed significantly as we have added extra birds to the LL analysis, following the reviewer's earlier suggestion. The values reported are now as the mean ± SEM, specifically for the 3 birds analyzed in both LD and LL. These 3 birds averaged a TST of 44.7% in LD conditions. This was very close to the overall 47.35% TST measured across all five birds.

3.12

p. 13, ll. 259-260
"For this reason, we chose to analyze the other LL birds with automated methods"
What means "automated methods" in this context? Do these methods only automatically analyze videos or are they only/also based on PSG data?

We had originally conducted automated motion data on the video data, but have now added automated PSG analysis of spectral charateristics (Figure 9f-i), following the reviewer's suggestions above. We have clarified the wording of this section to reflect this.

3.13

p. 13, ll. 266-267
"The response to constant light was somewhat individualized; for example, one bird (Bird 3) showed signs of free-running during the first few days of LL"
How did the other birds behave making their behavior individual?

We have added a short description of the other two LL birds, clarifying that free-running was less apparent in Birds 2 and 4, and commenting on other idiosyncratic reactions e.g. hyperactivity in Bird 2. We also show the behavioral scores of all three birds in LD vs LL day 8, as Figure S3.

3.14

p. 15, l. 301-302
"The duration of NREM episodes reached a peak in the second hour of the night…"
Is this peak statistically significant?

We applied the regression analysis from Figure 2d to Figures 3a-d in order to test for a significant change in the durations and frequencies of each sleep stage across the night. We have also added a figure showing the hourly TST average (Figure 4d). Birds tended to spend more time awake in the first hour of the night, then sleeping more consistently starting in hour 2. Comparing this to Figure 2d, this suggests this TST increase, along with the low %REM in hour 2, is responsible for the peak in NREM episode duration.

3.15

Fig. 3a
How does the shown video scoring compare with scoring based on PSG data?

Because the video was our primary means of distinguishing sleep from wake, in general the scoring changed very little during subsequent PSG scoring. We have added a

section to the results reporting on the epochs that differed between video and PSG scoring; for details see above response to comment #4.

3.16

p.15, l. 303
(Figure 3b) -> (Figure 4b)

Done.

3.17

Fig. 4D
Why negative Zeitgeber times?

We have changed the labeling to positive Zeitgeber times to maintain consistency across figures.

3.18

p. 16, ll 333-334
“We conclude that budgerigars exhibit extensive REM episodes characterized by a prevalence of high frequency peaks.“
How much does REM differ from Wake using delta and gamma power?

We have added spectral analysis of quiet wakefulness, comparing it with REM, IS and SWS (Figure 4 a-c; Figure S2a). In general wake is very similar to REM in the delta and gamma bands, but has higher power on average in the 4 – 16 Hz band.

3.19

p. 16, l. 335
“Spectral analyses confirm our manual classification of mixed-frequency IS interspersed with low-frequency SWS.”
Points for IS and SWS largely overlap along the y-axis in Fig. 4d. A more thorough analysis (see comments above) is required to establish that IS and SWS correspond to two distinct states. For example, is the distribution of delta and/or gamma power bimodal with the two modes corresponding to IS and SWS?

We have added histograms showing the distribution of the points in this figure (now Figure 5a), as well as a summary figure of the average distributions of delta and the number of peaks (a measure similar to gamma, but independent of spectral analysis) across the three sleep states in all five birds (Figure 4f-g).

3.20
p. 17, ll. 364-365
"Eye movements were detected as the anticorrelation of the left and right EOGs (Figure 5c)" -> Figure 5d

Done.

3.21
Fig. 6a
The 34 min oscillation is obvious and convincing in the example gamma/delta data. But the 1min and especially 10s oscillation is hard to see. The authors could show a zoom-in to make these faster oscillations more obvious and/or show that a powerspectrum of the gamma/delta signal has peaks at frequencies 1/60 and 1/10 Hz.

We agree it would be useful to see the faster rhythm, which is not visible in Fig 6a. We have added plots of the 10-s moving average at two different times of night, demonstrating the ~60s oscillation (Figure 6b).

3.22
p.18, l. 378
"reported in some other animals [50,51]"
Another example for a slow rhythm in mammals:
Lecci et al., "Coordinated Infraslow Neural and Cardiac Oscillations Mark Fragility and Offline Periods in Mammalian Sleep." Science Advances 3, no. 2 (2017). https://doi.org/10.1126/sciadv.1602026.

We thank the reviewer for the helpful reference; it has been added to the paper.

3.23
p. 23, l. 482
"Perhaps under normal LD, parrots on average exhibit a great deal of REM."
parakeets in this context, instead of parrots?

Done.

REFERENCES

1. Ayala-Guerrero F. Sleep patterns in the parakeet Melopsittacus undulatus. Physiol Behav. 1989;46: 787–791.

2. Tisdale RK, Vyssotski AL, Lesku JA, Rattenborg NC. Sleep-Related Electrophysiology and Behavior of Tinamous (Eudromia elegans): Tinamous Do Not Sleep Like Ostriches. Brain Behav Evol. 2017;89: 249–261. doi:10.1159/000475590

3. Lesku JA, Meyer LCR, Fuller A, Maloney SK, Dell'Omo G, Vyssotski AL, et al. Ostriches sleep like platypuses. PLoS ONE. Public Library of Science; 2011;6: e23203. doi:10.1371/journal.pone.0023203

4. van Hasselt SJ, Rusche M, Vyssotski AL, Verhulst S, Rattenborg NC, Meerlo P. The European starling (Sturnus vulgaris) shows signs of NREM sleep homeostasis but has very little REM sleep and no REM sleep homeostasis. Sleep. 2020;43: 269. doi:10.1093/sleep/zsz311

5. Tisdale RK, Lesku JA, Beckers GJL, Vyssotski AL, Rattenborg NC. The low-down on sleeping down low: pigeons shift to lighter forms of sleep when sleeping near the ground. J Exp Biol. The Company of Biologists Ltd; 2018;221: jeb182634. doi:10.1242/jeb.182634

6. Scriba MF, Henry I, Vyssotski AL, Mueller JC, Rattenborg NC, Roulin A. Ultradian Rhythmicity in Sleep-Wakefulness Is Related to Color in Nestling Barn Owls. J Biol Rhythms. SAGE PublicationsSage CA: Los Angeles, CA; 2017;32: 456–468. doi:10.1177/0748730417722250

7. Rattenborg NC, Voirin B, Cruz SM, Tisdale R, Dell'Omo G, Lipp H-P, et al. Evidence that birds sleep in mid-flight. Nat Comms. Nature Publishing Group; 2016;7: 12468. doi:10.1038/ncomms12468

8. Mexicano G, Montoya-Loaiza B, Ayala-Guerrero F. Sleep characteristics in the quail Coturnix coturnix. Physiol Behav. 2014;129: 167–172. doi:10.1016/j.physbeh.2014.02.041

9. Scriba MF, Ducrest A-L, Henry I, Vyssotski AL, Rattenborg NC, Roulin A. Linking melanism to brain development: expression of a melanism-related gene in barn owl feather follicles covaries with sleep ontogeny. Front Zool. BioMed Central; 2013;10: 42. doi:10.1186/1742-9994-10-42

10. Low PS, Shank SS, Sejnowski TJ, Margoliash D. Mammalian-like features of sleep structure in zebra finches. Proc Natl Acad Sci USA. 2008;105: 9081–9086. doi:10.1073/pnas.0703452105

11. Martinez-Gonzalez D, Lesku JA, Rattenborg NC. Increased EEG spectral power density during sleep following short-term sleep deprivation in pigeons (Columba livia): evidence for avian sleep homeostasis. Journal of Sleep Research. 2nd ed. 2008;17: 140–153. doi:10.1111/j.1365-2869.2008.00636.x

12. Jones SG, Vyazovskiy VV, Cirelli C, Tononi G, Benca RM. Homeostatic regulation of sleep in the white-crowned sparrow (Zonotrichia leucophrys gambelii). BMC Neurosci. 2008;9: 47–14. doi:10.1186/1471-2202-9-47

13. Fuchs T, Siegel JJ, Burgdorf J, Bingman VP. A selective serotonin reuptake inhibitor reduces REM sleep in the homing pigeon. 2006;87: 575–581. doi:10.1016/j.physbeh.2005.12.003

14. Rattenborg NC, Mandt BH, Obermeyer WH, Winsauer PJ, Huber R, Wikelski M, et al. Migratory sleeplessness in the white-crowned sparrow (Zonotrichia leucophrys gambelii). PLoS Biol. 2004;2: 0924–0936. doi:10.1371/journal.pbio.0020212

15. Ayala-Guerrero F, Mexicano G, Ramos JI. Sleep characteristics in the turkey Meleagris gallopavo. Physiol Behav. 2003;78: 435–440.

16. Szymczak JT, Kaiser W, Helb HW, Beszczyńska B. A study of sleep in the European blackbird. Physiol Behav. 1996;60: 1115–1120. doi:10.1016/0031-9384(96)00231-4

17. Berger RJ, Phillips NH. Constant light suppresses sleep and circadian rhythms in pigeons without consequent sleep rebound in darkness. Am J Physiol. 1994;267: R945–52.

18. Schmidt DF. A Comparative Analysis of Avian Sleep. Amlaner CJ Jr., editor. Doctoral dissertation, University of Arkansas. 1994.

19. Szymczak JT, Helb HW, Kaiser W. Electrophysiological and behavioral correlates of sleep in the blackbird (Turdus merula). Physiol Behav. 1993;53: 1201–1210. doi:10.1016/0031-9384(93)90380-X

20. Szymczak JT. Influence of environmental temperature and photoperiod on temporal structure of sleep in corvids. Acta Neurobiol Exp (Wars). 1989;49: 359–366.

21. Ayala-Guerrero F, Pérez MC, Calderón A. Sleep patterns in the bird Aratinga canicularis. Physiol Behav. 1988;43: 585–589.

22. Ayala-Guerrero F, Vasconcelos-Dueñas I. Sleep in the dove Zenaida asiatica. Behavioral and Neural Biology. 1988;49: 133–138. doi:10.1016/S0163-1047(88)90451-7

23. Tobler I, Borbély AA. Sleep and EEG spectra in the pigeon (Columba livia) under baseline conditions and after sleep deprivation. J Comp Physiol. Springer-Verlag; 1988;163: 729–738. doi:10.1007/BF00604050

24. Szymczak JT. Daily distribution of sleep states in the rook Corvus frugilegus. J Comp Physiol A. 1987;161: 321–327.

25. van Luijtelaar EL, van der Grinten CP, Blokhuis HJ, Coenen AM. Sleep in the domestic hen (Gallus domesticus). Physiol Behav. 1987;41: 409–414.

26. Buchet C, Dewasmes G, Le Maho Y. An electrophysiological and behavioral study of sleep in emperor penguins under natural ambient conditions. Physiol Behav. 1986;38: 331–335. doi:10.1016/0031-9384(86)90103-4

27. Szymczak JT. Daily distribution of sleep states in the jackdaw, Corvus monedula. Chronobiologia. 1986;13: 227–235.

28. Szymczak JT. Daily rhythm of sleep-wakefulness in the starling, Sturnus vulgaris. Acta Physiol Pol. 1986;37: 199–206.

29. Szymczak JT. Sleep pattern in the starling (Sturnus vulgaris). Acta Physiol Pol. 1985;36: 323–331.

30. Dewasmes G, Cohen-Adad F, Koubi H, Le Maho Y. Polygraphic and behavioral study of sleep in geese: existence of nuchal atonia during paradoxical sleep. Physiol Behav. 1985;35: 67–73. doi:10.1016/0031-9384(85)90173-8

31. Stahel CD, Megirian D, Nicol SC. Sleep and metabolic rate in the little penguin, Eudyptula minor. J Comp Physiol B. Springer-Verlag; 1984;154: 487–494. doi:10.1007/BF02515153

32. Walker LE, Walker JM, Palca JW, Berger RJ. A continuum of sleep and shallow torpor in fasting doves. Science. 1983;221: 194–195. doi:10.1126/science.221.4606.194

33. Speciale SG Jr, Nowaczyk T, Jouvet M. Chick phasic bioelectric activity at the time of hatching and the effects of previous nialamide injection. Brain Res. Elsevier; 1976;101: 148–154. doi:10.1016/0006-8993(76)90996-3

34. Saucier D, Astic L. Etude polygraphique du sommeil chez le poussin a l'eclosion. Evolution aux 3ème ET 4ème jours. Electroencephalography and Clinical Neurophysiology. 1975;38: 303–306. doi:10.1016/0013-4694(75)90251-5

35. Schlehuber CJ, Flaming DG, Lange GD, Spooner CE. Paradoxical sleep in the chick (Gallus domesticus). Behav Biol. 1974;11: 537–546.

36. Šušić VT, Kovačević RM. Sleep patterns in the owl Strix aluco. Physiol Behav. 1973;11: 313–317. doi:10.1016/0031-9384(73)90005-X

37. Karmanova IG, Churnosov EV. Electrophysiological investigation of natural sleep and waking in turtles and hens. Neurosci Behav Physiol. Kluwer Academic Publishers; 1973;6: 83–90. doi:10.1007/BF01186007

38. Sugihara K, Gotoh J. Depth-electroencephalograms of chickens in wakefulness and sleep. Jpn J Physiol. 1973;23: 371–379. doi:10.2170/jjphysiol.23.371

39. Berger RJ, Walker JM. Sleep in the burrowing owl (Speotyto cunicularia hypugaea). Behav Biol. 1972;7: 183–194.

40. Walker JM, Berger RJ. Sleep in the domestic pigeon (Columba livia). Behav Biol. 1972;7: 195–203. doi:10.1016/S0091-6773(72)80199-8

41. Van Twyver H, Allison T. A polygraphic and behavioral study of sleep in the pigeon (Columba livia). Experimental Neurology. 1972;35: 138–153. doi:10.1016/0014-4886(72)90065-9

42. Rojas-Ramírez JA, Tauber ES. Paradoxical Sleep in Two Species of Avian Predator (Falconiformes). Science. American Association for the Advancement of Science; 1970;168: 956–956. doi:10.1126/science.168.3934.956-c

43. Hishikawa Y, Cramer H, Kuhlo W. Natural and melatonin-induced sleep in young chickens? A behavioral and electrographic study. Exp Brain Res. 1969;7: 84–94. doi:10.1007/BF00236109

44. Tradardi V. Sleep in the pigeon. Arch Ital Biol. 1966;104: 516–521.

45. Ookawa T, Gotoh J. Electroencephalogram of the chicken recorded from the skull under various conditions. Journal of Comparative Neurology. Wiley Subscription Services, Inc., A Wiley Company; 1965;124: 1–14. doi:10.1002/cne.901240102

46. Ookawa T, Gotoh J. Electroencephalographs Study of Chickens: Periodic Recurrence of Low Voltage and Fast Waves during Behavioral Sleep. Poultry Science. Oxford University Press; 1964;43: 1603–1604. doi:10.3382/ps.0431603

47. Klein M, Jouvet M, Michel F. Etude polygraphique du sommeil chez les oiseaux. Paris; 1964. pp. 99–103. Available: https://sommeil.univ-lyon1.fr/articles/jouvet/crssb_63/print.php

48. Brockway BF. Ethological Studies of the Budgerigar (Melopsittacus Undulatus): Non-Reproductive Behavior. Behaviour. 1964;22: 193–222. doi:10.1163/156853964X00021

49. Dooling RJ. Behavioral audiometry with the parakeet Melopsittacus undulatus. The Journal of the Acoustical Society of America. 1973;53: 1757–1758. doi:10.1121/1.1913538

50. Brauth SE, McHale CM, Brasher CA, Dooling RJ. Auditory pathways in the budgerigar. I. Thalamo-telencephalic projections. Brain Behav Evol. Karger Publishers; 1987;30: 174–199. doi:10.1159/000118645

51. Okanoya K, Dooling RJ. Detection of gaps in noise by budgerigars (Melopsittacus undulatus) and zebra finches (Poephila guttata). Hear Res. 1990;50: 185–192. doi:10.1016/0378-5955(90)90044-p

52. Hall WS, Brauth SE, Heaton JT. Comparison of the effects of lesions in nucleus basalis and field "L" on vocal learning and performance in the budgerigar (Melopsittacus undulatus). Brain Behav Evol. Karger Publishers; 1994;44: 133–148. doi:10.1159/000113585

53. Striedter GF. The vocal control pathways in budgerigars differ from those in songbirds. Wiley-Liss, Inc; 1994;343: 35–56. doi:10.1002/cne.903430104

54. Dooling RJ, Ryals BM, Manabe K. Recovery of hearing and vocal behavior after hair-cell regeneration. PNAS. National Academy of Sciences; 1997;94: 14206–14210. doi:10.1073/pnas.94.25.14206

55. Durand SE, Heaton JT, Amateau SK, Brauth SE. Vocal control pathways through the anterior forebrain of a parrot (Melopsittacus undulatus). J Comp Neurol. John Wiley & Sons, Ltd; 1997;377: 179–206. doi:10.1002/(sici)1096-9861(19970113)377:2<179::aid-cne3>3.0.co;2-0

56. Shea SD, Heaton KJ, Heaton JT, Hall WS, Brauth SE. The role of contact calls in the social behavior of the budgerigar (Melopsittacus undulatus). Annals of the New York Academy of Sciences. John Wiley & Sons, Ltd; 1997;807: 571–573. doi:10.1111/j.1749-6632.1997.tb51971.x

57. Jarvis ED, Mello CV. Molecular mapping of brain areas involved in parrot vocal communication. John Wiley & Sons, Inc; 2000;419: 1–31. doi:10.1002/(sici)1096-9861(20000327)419:1<1::aid-cne1>3.0.co;2-m

58. Hile AG, Burley NT, Coopersmith CB, Foster VS, Striedter GF. Effects of Male Vocal Learning on Female Behavior in the Budgerigar, Melopsittacus undulatus. Ethology. 2005;111: 901–923. doi:10.1111/j.1439-0310.2005.01105.x

59. Osmanski MS, Dooling RJ. The effect of altered auditory feedback on control of vocal production in budgerigars (Melopsittacus undulatus). The Journal of the Acoustical Society of America. 2009;126: 911–919. doi:10.1121/1.3158928

60. Eda-Fujiwara H, Imagawa T, Matsushita M, Matsuda Y, Takeuchi H-A, Satoh R, et al. Localized brain activation related to the strength of auditory learning in a parrot. Hausberger M, editor. PLoS ONE. 2012;7: e38803. doi:10.1371/journal.pone.0038803

61. Tu H-W, Dooling RJ. Perception of warble song in budgerigars (Melopsittacus undulatus): evidence for special processing. Anim Cogn. 2nd ed. Springer-Verlag; 2012;15: 1151–1159. doi:10.1007/s10071-012-0539-1

62. Garcia-Calero E, Bahamonde O, Martinez S. Differences in number and distribution of striatal calbindin medium spiny neurons between a vocal-learner (Melopsittacus undulatus) and a non-vocal learner bird (Colinus virginianus). Front Neuroanat. Frontiers; 2013;7: 46. doi:10.3389/fnana.2013.00046

63. Dahlin CR, Young AM, Cordier B, Mundry R, Wright TF. A test of multiple hypotheses for the function of call sharing in female budgerigars, Melopsittacus undulatus. Behav Ecol Sociobiol (Print). Springer Berlin Heidelberg; 2014;68: 145–161. doi:10.1007/s00265-013-1631-5

64. Hara E, Perez JM, Whitney O, Chen Q, White SA, Wright TF. Neural FoxP2 and FoxP1 expression in the budgerigar, an avian species with adult vocal learning. Elsevier B.V; 2015;283: 1–8. doi:10.1016/j.bbr.2015.01.017

65. Spierings MJ, Cate Ten C. Budgerigars and zebra finches differ in how they generalize in an artificial grammar learning experiment. Proc Natl Acad Sci USA. National Acad Sciences; 2016;113: E3977–84. doi:10.1073/pnas.1600483113

66. Seki Y, Dooling RJ. Effect of auditory stimuli on conditioned vocal behavior of budgerigars. Behav Processes. 2016;122: 87–89. doi:10.1016/j.beproc.2015.11.002

67. Chen J, Zou Y, Sun Y-H, Cate Ten C. Problem-solving males become more attractive to female budgerigars. Science. American Association for the Advancement of Science; 2019;363: 166–167. doi:10.1126/science.aau8181

68. Yu AC, Margoliash D. Temporal Hierarchical Control of Singing in Birds. Science. American Association for the Advancement of Science; 1996;273: 1871–1875. doi:10.1126/science.273.5283.1871

69. Buckley DP, Duggan MR, Anderson MJ. Budgie in the mirror: An exploratory analysis of social behaviors and mirror use in the Budgerigar (Melopsittacus undulatus). Behav Processes. 2017;135: 66–70. doi:10.1016/j.beproc.2016.11.015

70. Buckelmüller J, Landolt HP, Stassen HH, Achermann P. Trait-like individual differences in the human sleep electroencephalogram. Neuroscience. 2006;138: 351–356. doi:10.1016/j.neuroscience.2005.11.005

71. Ohayon MM, Carskadon MA, Guilleminault C, Vitiello MV. Meta-analysis of quantitative sleep parameters from childhood to old age in healthy individuals: developing normative sleep values across the human lifespan. Sleep. 2004;27: 1255–1273.

72. Rechtschaffen A, Kales A. A manual of standardized, techniques and scoring system for sleep stages of human sleep. Los Angeles: Brain Information Service. Brain Research Institute; 1968.

73. Ancoli-Israel S, Chesson A, Quan SF. The AASM manual for the scoring of sleep and associated events: rules, terminology and technical specifications. American Academy of Sleep Medicine; 2007. doi:10.1111/j.1365-2869.2005.00474.x/full

74. Roth TC, Lesku JA, Amlaner CJ, Lima SL. A phylogenetic analysis of the correlates of sleep in birds. Journal of Sleep Research. 2006;15: 395–402. doi:10.1111/j.1365-2869.2006.00559.x

75. Kis A, Szakadát S, Gácsi M, Kovács E, Simor P, Török C, et al. The interrelated effect of sleep and learning in dogs (Canis familiaris); an EEG and behavioural study. Sci Rep. Nature Publishing Group; 2017;7: 41873. doi:10.1038/srep41873
