# Supplementary material for: Budgerigars have complex sleep structure similar to that of mammals
Source: PLoS Biol. 2020 Nov 17;18(11):e3000929. doi: 10.1371/journal.pbio.3000929 (PMC7707536; doi:10.1371/journal.pbio.3000929)
Supplement: Attachment — Submitted filename: Response to reviewer.docx [file pbio.3000929.s011.docx]

Thank you for these additional clarifying comments.

Reviewer #3:

*Overall, the authors well addressed all of my concerns. Here some remaining final questions/ concerns about the new analyses:*

**Fig. 3**
*The transition matrix in Fig. 3h is extremely helpful in understanding the dynamics of the sleep pattern.
I'm still a bit puzzled about what the edges in the chord diagrams represent. Fig. 3f contains a single edge that represents both IS -> REM and REM -> IS?
Naively, I would have assumed that all the outgoing edges of one node (state) correspond to one row in the transition matrix (which sums up to 100%). That is, the outgoing edges show the likelihood to go to either of the states (P[current state -> next state])*

This is correct. We have added a label to Fig 3f (REM 🡪 SWS) to help clarify how the bidirectional edges work. We have also adjusted the wording of the caption to better explain this.

*Minor: Some of the rows in the matrix don't sum up to 100%, which is probably the result of rounding errors.*

This is correct as well. The numbers in Fig 3h were each rounded to integers to reduce visual clutter. We now note this in the legend.

**Fig. 9**
*(c) Why are there no error bars for SWA under LD conditions? What's the difference to the SWA data shown in Fig. 4d (which does have error bars, but seems to be slightly different)?*

We have added error bars to the LD line in Fig 9f-g. The SWA in Fig 4d is calculated from NREM epochs only, to remain consistent with the typical definition of SWA in the field. The SWA in Fig 9f, however, is calculated for all sleep epochs to allow comparison between LD and LL (for which REM vs NREM has not been scored). We have adjusted the caption to highlight this distinction.

*(c) What's the REM latency under LL?*

The LL REM latency is unknown, as we did not manually score the LL PSG but instead chose to use automated/unbiased methods of analysis. Given the data shown in Fig 9c, this leaves two options: either 1) the REM latency in LL is the same as in LD, in which case there are likely fewer opportunities for REM sleep and/or they are cut short; or 2) the REM latency in LL is shorter, which would suggest highly disrupted sleep architecture (ie REM rebound) and curtailing of NREM episodes.

*What means 'pd' in 'LD mean sleep pd duration'?*

This was an abbreviation for “sleep period”; however, we realize this is confusing given the use of the word “period” elsewhere in the figure. We have changed the x-axis labels in Fig 9c to “sleep episode duration.”

**Fig. 6e:**
*What means the y-axis? I don't understand the "40% REM", "100% IS", and "20% SWS"? Would it be possible to plot for each state a separate line showing its rate or probability as function of the phase?*

We agree this suggestion is more intuitive and have replaced Fig 6e with plots showing % REM and % SWS as a function of phase.

**Fig. 8**
*What about PC1 vs. PC3 (or PC2); do the data points for REM, IS, SWS lie along PC1 as in Low et al. 2008?*

We have added the PC plots for all five birds as a new supplementary figure (Fig S4).

*There are some "left over fonts" in (b, c) between "delta" and "-4".*

Fixed.

*And quite a few points are located outside the 3D axes.*

This is correct; however, we thought it best to show these points rather than exclude them, in order to acknowledge that these points exist. We also chose not to expand the axis limits to encompass all of these points: the axis limits are set to match those in Low 2008 and work best for displaying the pattern of interest.

**Miscellaneous:**
*-Line 1276: "frequency bins with p > 0.05 were identified." Do you mean p < 0.05?*

Yes – thank you for catching this; this has been corrected.

*-Line 573: "many epochs identified as IS that did not collapse with either treatment?" What treatment?*

The “treatment” refers to the different three-dimensional spaces used to plot the points. This line has been revised to clarify this.

*-What is the rate of false positives and false negatives for REM, IS, SWS when comparing manual and automatic scoring? It may provide a more intuitive measure for how well manual and automatic scoring match.*

We have added sensitivity (i.e. true positives/all positives) and specificity (i.e. true negatives/all negatives) for REM and SWS detection to the Methods.
